# A data-driven global observatory addressing worldwide challenges through text mining and complex data visualisation

**DOI:** 10.12688/openreseurope.14471.2

**Published:** 2023-12-07

**Authors:** Joao Costa, M. Besher Massri, Marko Grobelnik, Ignacio Casals del Busto, Dale Weston

**Affiliations:** 1 Quintelligence, Ljubljana, Slovenia; 2 UNESCO International Research Institute on AI - IRCAI, Ljubljana, Slovenia; 3 Institute Jozef Stefan, Ljubljana, Slovenia; 4 Aguas del Alicante, Alicante, Spain; 5 Public Health England, Salisbury, UK

**Keywords:** Big Data, Semantic Technologies, Public Health, Water Management, Text Mining, MeSH Headings, MEDLINE, multilingual news, Social media

## Abstract

Observing the world on a global scale can help us understand better the context of problems that engage us all. In this paper, we propose a data-driven global observatory methodology that puts together the different perspectives of media, science, statistics and sensing over heterogeneous data sources and text mining algorithms. We also discuss the implementation of this global observatory in the context of epidemic intelligence, monitoring the impact of the COVID-19 pandemic, and in the context of climate change, with a specific focus on water resource management. Moreover, we discuss the value of this global solution in local contexts and priorities, based on the exchange with stakeholders in municipalities, utilities and governmental institutions.

## Plain language summary

In this research paper we lay the foundations for a data-driven perspective on how to appropriately use open data to build business intelligence, with proven usefulness in applications to public health and water resource management and with potential applications to other priority areas.

## II. Introduction

The world’s globalization phenomena unveiled awareness of worldwide problems, such as the climate crisis, but also to common efforts to find solutions to those problems, as was the case of the several COVID-19 collaborative actions. There are many obstacles still today on such global strategies to which innovative technology and data-driven solutions can help. In this paper we propose the concept of a global observatory based on text mining algorithms that is able to answer the wide range questions that are core to global solutions, using machine learning and Big Data analytic methods over the layered information it is ingesting often in real-time. The main perspectives of this global observatory fall on: (i) the monitoring and exploration of news articles and social media feeds; (ii) the analysis of combinations of indicators through time and what stories can they tell; and (iii) the exploration of published scientific knowledge. All of these perspectives can be combined to provide complementary answers to main topics from health to engineering. In this paper we discuss the results obtained based on two implementations of these observatory approach: (a) the Coronavirus Watch portal released in 1 addressing the worldwide spread of COVID-19 ^2^; and (b) the NAIADES Water Observatory ^3^, focusing on best practices to build water sustainability.

## III. Methods

**Figure 1.**
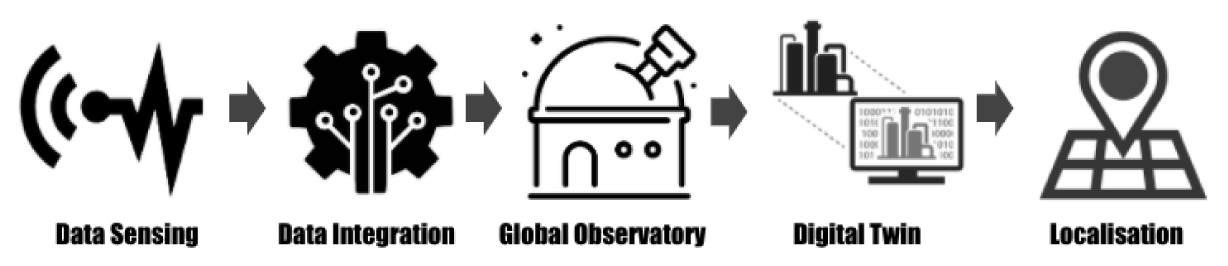
The approach used leading from data sensing to the digital twin and its approximation to local priorities.

Taking into account the schema in Figure 1, we consider the construction of the Global Water Observatory into phases, going from lower to higher complexity. A similar observatory, dedicated to monitor COVID-19 ^2^, was made available with less functionality but also including a diversity of perspectives for which the interoperability is a core topic of discussion in this paper. We start by putting together data sources that are meaningful to a range of stakeholders targeted within engaged citizens to decision makers and that can leverage the information provided to established evidence-based policies.

At the data collection phase, we are concerned with addressing properly the challenges in the heterogeneous nature of the data, their different frequency and size, as well as the levels of access to it established by data providers. These parameters to take into consideration ensure the appropriate data ingestion into the system. The selection of data sources and features to be ingested is done manually, but the ingestion itself is automated when the frequency requires so. The frequency of update depends solely of the data provider. At this first stage we are collecting data from many different data sources (such as, *e.g.*, the Worldwide news, the Microsoft Academic Graph, the Word Bank, the United Nations Sustainable Development Goals), according to their relation to the focus topics and priorities.

A forthcoming stage is in the data cleaning, data processing and data integration prior ingestion. This step is highly important to allow for the data quality that is needed in order to obtain useful insights from it. In this step we include the data curation, where the most meaningful datasets are selected and parsed. We also include the exploratory data analysis and some data visualisation for the purpose of prototyping what is then available at the Water Observatory.

The Observatory phase is then possible when the curated data streams of a selection of dynamic data sources are live in the system and can be used to obtain insight on particular topics of interest, monitor Key Performance Indicators associated with business priorities, and allow for a global and local perspective on related topics. These include interactive data visualisations of indicators and statistical data, the dynamic view of the news sources over priorities, or the user query over a scientific research topic. This allows for insight on topics in analysis (such as water topics like, *e.g.*, water scarcity and water quality, and public health topics like, *e.g.*, ebola or the new coronavirus) that will be put into the context of local data when sourced from the shared interest of users.

The path ahead is a novel concept of a meaningful Digital Twin ( *i.e.*, a dynamical model which, given a current state of an observed system, is capable of a digital partial reconstruction of such a system) that builds over the Global Water Observatory to rise above data complexity towards data interoperability. This is usually difficult to achieve in full due to the heterogeneity of the data, the different characteristics of the data sourced (frequency, data types, *etc.*) and the domain knowledge needed to identify new challenges covering a wide range of business intelligence priorities. Nevertheless, useful aspects of it can be achieved, some of which are already evident from the implementations discussed in this paper. An example of this is to track a topic in the news, its impact in the social media, and explore the range of the problem in the published scientific research, as well as extract good practices to deal with this problem.

We add a final stage to this diagram that is usually forgotten in a theoretical framework, which is the adaptation of the system to the needs and priorities in the user side. Here we consider the ingestion of local data, the customization of news streams, the availability of exploratory dashboards, the shareable instances for policy makers, and the APIs for 3rd party integration.

The system that is able to access the data sources that relate to the items above, is also able to track the term throughout the several phases of popularization. It is also able to show the current status of a particular topic of interest, and optimally alert for potentially trendy topics in the future. In that particular context, the interactive data visualisation is a key factor to improve the usefulness of the tool and should express visual narratives that comprehend the relevant aspects of the problem. Good examples of such can be consulted for the case of epidemic intelligence in 4 and water intelligence in 5, and will be discussed in the implementations described and explored for the purpose of this study. 

## IV. On The Coronaviruswatch Observatory

When the World Health Organization (WHO) announced the global COVID-19 pandemic on March 11th 2020 ^6^, following the rising incidence of the SARS-CoV-2 in Europe, the world started reading and talking about the new Coronavirus. The arrival of the epidemic to Europe scaled out the news published about the topic, while public health institutions and governmental agencies had to look for existing reliable solutions that could help them plan their actions and the consequences of these.

Technological companies and scientific communities invested efforts in making available tools ( *e.g.* the GIS ^7^ later adopted by WHO), challenges ( *e.g.* the Kaggle COVID-19 competition ^8^), and scientific reports and data ( *e.g.* the repositories medRxiv ^9^ and Zenodo ^10^).

**Figure 2.**
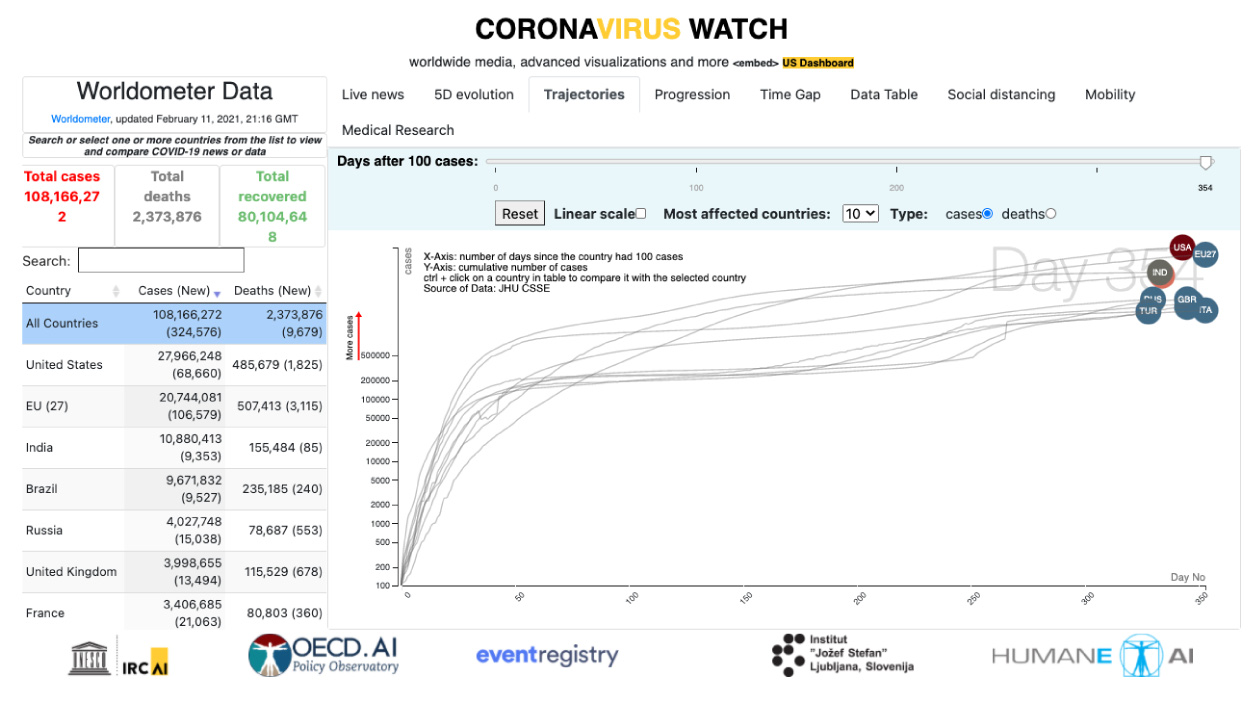
The IRCAI observatory for the COVID-19 pandemic.

In March 2020 we have released the first implementation of this global observatory as the Coronavirus Watch portal ^1^, aiming to contribute to a multinational response to the global crisis. It was made available by the UNESCO AI Research Institute (IRCAI), comprehending several data exploration dashboards related to the SARS-CoV-2 worldwide pandemic. This platform aimed to expose the different perspectives on the data generated and trigger actions that can contribute to a better understanding of the behavior of the disease (see Figure 2).

The portal includes a real-time news monitoring system that can be focused at European and national level, side-by-side with the data on the progress of the pandemics made available by the Worldometer ^11^ and the Center for Disease Control ^12^. The visual representation of the details of those indicators were made available over animations showcasing the live comparisons in 5D (as in Figure 2), the trajectories of the most affected countries, and the details of the progression of the disease. It also included perspectives on the mobility, sourced on the Google Community mobility data ^13^, a social distancing simulator, and exploration tools based on the published biomedical research (see 2).

**Figure 3.**
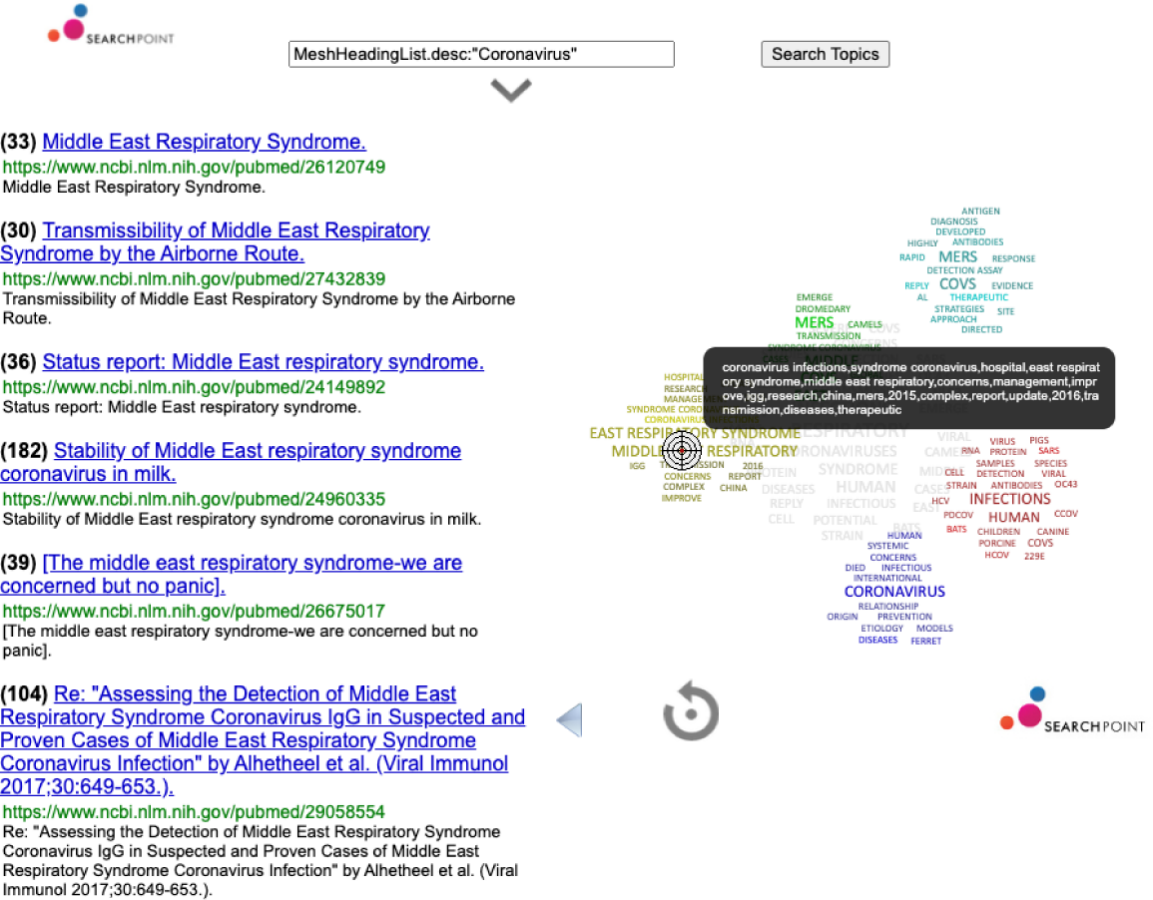
Exploring scientific research through complex data visualisation.

To improve the resolution of these results and to optimise their relevance for European public health agencies, we have developed a set of COVID-19 focused tools on the MIDAS platform ^14^. This system was designed for evidence-based decision-making in public health. This approach allows us to validate the usability of the global observatory on a cross-EU level within the COVID-19 context, integrating both health news and biomedical research exploration (see Figure 3).

## V. On The Water Observatory

Climate change is a global problem that in the recent years has been in the focus of European and Worldwide strategies. The priorities in European Union are rapidly changing towards sustainability and environmental efficiency, transversely to most domains of action. The European Commission’s Green Deal aiming for a climate neutral Europe in 2050, and boosting economy through green technology ^15^ provides a new framework to understand and position water resource management in the context of the challenges of tomorrow ^16^. In this context, the NAIADES project ^17^ aims to improve the water resource management in a global context, including European regions where water scarcity is predicted, also dealing with concerns as, *e.g.*, saline intrusion and groundwater contamination. To contribute to this cause, we deployed a Global Water Observatory ^3^ that is focusing on water-related aspects allowing the user to explore the several layers of information it is providing, from news and social media to published science, weather models and indicators. The NAIADES Global Water Observatory does not only contribute to the improvement of European sustainability in water-related matters, but also provides the local actors on the water resource management an active role in that taking into consideration the national and international open data available on water resource management-related topics and priorities (see 18 for more details).

Water is fundamental to all human activity and ecosystem health, and is a topic of rising awareness in the context of the recent discussions on climate change. Water resource management is central to those concerns, with the industry accounting for over 19% of global water withdrawal, and agricultural supply chains are responsible for 70% of water stress ^19^. In 2015 the UN established ”clean water and sanitation for all” as one of the 17 Sustainable Development Goals, aiming for eight targets to be achieved by 2030 ^20^. The UN secretary-general points out in April 2020 that SDG 6 is ”badly off track” compromising the progress on the 2030 Agenda ^21^. As noted by the Organisation for Economic Cooperation and Development (OECD), the ‘water crisis’ has often proven to be a crisis of governance ^22^, where water scarcity is largely caused by mismanagement of resources, leading to a global prioritisation ^23^.

The intention to globally monitor water resources is not new, and already in the late 1960s ^24^ the first spatially-distributed water resources model appeared, with first operational uses of satellite observations in water resources developed in the early 1980s ^25^. The reliable management of water resources is only possible under condition of availability of adequate qualitative and quantitative information about state of the water body at any moment of time. Taking advantage of the recent technological progress enabling much innovation that was unthinkable a few years ago, the concept of the Digital Twin is increasingly entering the water sector as an innovation driver. Due to the rapidly growing awareness of the sustainability challenges that we are facing in Europe and worldwide in the context of the water resource management, there has been much work done to develop systems that are able to collect information about the available water and even simulate and forecast that in the near future. These are usually geolocation-based systems ingesting water-related data to enable real-time monitoring of resources and usage ^26^. The other typical approach is the systems focused on workflows in the water sector, including the management of water distribution networks, hydraulic efficiency or leak/fraud detection, better suited to those companies that already have their infrastructure in place and know well what do they want to monitor ^27^.

The approach we proposed in this paper is novel in many ways. The news monitoring perspective is monitoring water scarcity and water quality in worldwide and, in particular, in the surrounding regions of the water resource management agencies is is mainly addressing, together with their audiences. This is also including a Twitter observatory that adds to the already implemented measure of impact of the monitored news in Facebook, a social media component to the observatory. The global indicators that are already available for visualisation, sourced over the UN Open Data Portal, the water-focused Sustainable Development Goal 6, and the World Bank Data Portal, can help us understand water-related aspects of the climate crisis.

The important role of scientific research in this context, and the best practices that can be extracted from this data, is explored with a complex data visualisation technology that allows the user to powerful Lucene-based queries over the article’s metadata aiming to refine search by moving a pointer over clusters of related topics (see Figure 3). We will also be including other data analytics technologies to analyse simultaneously multiple time-series providing interactive exploration tools to understand trends in the weather and water-related impacts to it.

The localisation of this global system entails the customisation of its functionality in news monitoring, ingestion of local indicators and exploration of scientific research on observed problems in, *e.g.*, groundwater contamination. In that, the observatory is synchronising with the priorities of regional water providers. These agencies ( *e.g.* Aguas de Alicante) are collecting data on their water resource management services to improve the customer satisfaction and optimise their system.

## VI. Conclusions and future work

The results discussed in this paper show the potential impact of the proposed data-driven global observatory in contexts like public health and climate change preparedness. This integrated system is capable to monitor in real-time the worldwide news and social media, statistics, published science, weather and many other data streams that are identified as useful and can be provide complementary value to those considered already. We will be deploying this system in the context of other global problems where there is enough data to provide useful and meaningful contribution, either in other aspects of the climate crisis to better plan response, in addressing other epidemiological concerns to serve as early warning, or in addressing a new focus in the context of data science for social good.

We are now working on extending this system to integrate the information retrieved by the topics searched over the internet provided by Google Trends, regarding issues related to the context in focus. The user will be able to explore a wide range of indicators and compare trends in a global and local level throughout a meaningful timeline. We will also be reusing EC-funded open datasets and initiatives in order to ingest this information as European-level indicators to complement the analysis. Furthermore, we will be further investigating the validity of the localization of this Global Water Observatory, integrating some of the local data that can be provided by the user, and customizing news sources to their own priorities, as well as making available data exploration dashboards that allow for further insight and evidence-based policy.

## VIII. Ethics and consent

Ethical approval and consent were not required

## Data Availability

For this paper, we used only open data. In particular, we have used the MEDLINE dataset ^28^ and the worldwide news are being collected by 29, freely available online, but which the dataset we do not have permission to share.
