# Supplementary material for: A data-driven global observatory addressing worldwide challenges through text mining and complex data visualisation
Source: Open Res Eur. 2023 Dec 7;2:68. Originally published 2022 May 30. [Version 2] doi: 10.12688/openreseurope.14471.2 (PMC11362724; doi:10.12688/openreseurope.14471.2)
Supplement: Peer review history

# Reviewer report: Reviewer response for version 2

**DOI:** 10.21956/openreseurope.18280.r47777

**Published:** 2025-01-09

**Authors:** Ángel M. García-Vico

**Affiliations:** 1 University of Jaén, Jaén, Andalusia, Spain

This paper I have been asked to review presents a data-driven global observatory framework that leverages text mining and complex data visualization to address global challenges, specifically focusing on epidemic intelligence and climate change (water resource management). The framework aggregates data from heterogeneous sources, such as media, scientific publications, and statistical indicators, to deliver actionable insights through real-time monitoring, analysis, and interactive dashboards. The paper in general is well-written and it is very easy to follow. Also, the proposal is interesting for the community. However, I have two main concerns about this work:

 1) While the conceptual framework is well-detailed, the technical specifics lack clarity. Can you detail the data cleaning and preprocessing pipeline? How do you handle issues like data bias, missing data, or noise?

 2) How does this work compare to other global observatories? For example, how does it outperform or complement existing systems like WHO dashboards or NASA’s climate monitoring systems?

Is the study design appropriate and does the work have academic merit?

Yes

Is the work clearly and accurately presented and does it cite the current literature?

Yes

If applicable, is the statistical analysis and its interpretation appropriate?

Not applicable

Are all the source data underlying the results available to ensure full reproducibility?

Yes

Are the conclusions drawn adequately supported by the results?

Yes

Are sufficient details of methods and analysis provided to allow replication by others?

Partly

Reviewer Expertise:

Computer Science, artificial intelligence

I confirm that I have read this submission and believe that I have an appropriate level of expertise to confirm that it is of an acceptable scientific standard, however I have significant reservations, as outlined above.

# Reviewer report: Reviewer response for version 2

**DOI:** 10.21956/openreseurope.18280.r42254

**Published:** 2024-08-29

**Authors:** Farah Jemili

**Affiliations:** 1 Mars Research Laboratory, ISITCOM, Universite de Sousse, Sousse, Sousse, Tunisia

Strengths:

1. The paper introduces a novel, data-driven global observatory that effectively integrates text mining with complex data visualization to address global challenges. This approach is well-grounded in addressing critical issues like epidemic intelligence and water resource management.
2. The paper meticulously outlines the stages of constructing the global observatory, from data collection to real-time monitoring and visualization, ensuring clarity in how the system operates.
3. The implementation examples, such as the Coronavirus Watch portal and the NAIADES Water Observatory, provide concrete evidence of the system's applicability and effectiveness in real-world scenarios.

Areas for Improvement:

1. Some sections, particularly the methods and implementation, could benefit from clearer language and more concise explanations. Reducing jargon and complex sentences would make the content more accessible.
2. While improvements have been made, the paper could further enhance the discussion on how this work compares with existing solutions, particularly in the areas of text mining and data visualization in global observatories.
3. The paper discusses the system's potential for customization based on local priorities, but more details on how this adaptation is practically implemented would strengthen the argument for its usability and flexibility.

Specific Suggestions:

- **Abstract**: Consider summarizing the key findings more explicitly to highlight the impact of the proposed observatory.
- **Figures**: Ensure that all figures are clearly labeled and referenced in the text to aid in the reader's understanding.
- **Conclusion**: Expand on the implications of your findings for future research and potential broader applications beyond the discussed domains.

Is the study design appropriate and does the work have academic merit?

Partly

Is the work clearly and accurately presented and does it cite the current literature?

Partly

If applicable, is the statistical analysis and its interpretation appropriate?

No

Are all the source data underlying the results available to ensure full reproducibility?

Partly

Are the conclusions drawn adequately supported by the results?

Partly

Are sufficient details of methods and analysis provided to allow replication by others?

Partly

Reviewer Expertise:

AI and Big Data

I confirm that I have read this submission and believe that I have an appropriate level of expertise to confirm that it is of an acceptable scientific standard, however I have significant reservations, as outlined above.

# Reviewer report: Reviewer response for version 2

**DOI:** 10.21956/openreseurope.18280.r36649

**Published:** 2024-01-02

**Authors:** Carson Leung

**Affiliations:** 1 University of Manitoba, Manitoba, Canada

Costa et al. made some revisions based on previous peer review reports. They described in this 7-page revised submission a data-driven global observatory addressing two worldwide challenges (namely, public health and water) through text mining and complex data visualisation. They focused on using open data appropriately to monitor news articles and social media feeds related to COVID-19 and water shortage. They would collect, clean, process, integrate and ingest data by making use of key performance indicators (KPI) and digital twin. The presented idea is interesting.

Is the study design appropriate and does the work have academic merit?

Yes

Is the work clearly and accurately presented and does it cite the current literature?

Partly

If applicable, is the statistical analysis and its interpretation appropriate?

Not applicable

Are all the source data underlying the results available to ensure full reproducibility?

Yes

Are the conclusions drawn adequately supported by the results?

Partly

Are sufficient details of methods and analysis provided to allow replication by others?

Partly

Reviewer Expertise:

Big data, public health, COVID-19, text mining, social media, data visualisation, data science

I confirm that I have read this submission and believe that I have an appropriate level of expertise to confirm that it is of an acceptable scientific standard.

# Reviewer report: Reviewer response for version 1

**DOI:** 10.21956/openreseurope.15624.r32818

**Published:** 2023-07-10

**Authors:** Carson Leung

**Affiliations:** 1 University of Manitoba, Manitoba, Canada

Costa et al. described in this 8-page submission a data-driven global observatory addressing two worldwide challenges (namely, public health and water) through text mining and complex data visualisation. They focused on using open data appropriately to monitor news articles and social media feeds related to COVID-19 and water shortage. They would collect, clean, process, integrate and ingest data by making use of KPI and digital twin.

 The presented idea is interesting. It would be nice if the authors could consider presenting the work more clearly and providing more details, especially for the corona virus watch observatory. For instance, the authors briefly described how to monitor news articles and social media feeds related to water shortage and quality. However, shorter descriptions were provided for COVID-19.

 It would also be nice if the authors could consider providing more details of methods and analysis to allow replication by others. For instance, it is unclear what text mining techniques or what complex data visualisation was used.

 Out of the list 26 references, only 5 were from journals. It would be nice if the authors could consider citing some more current formal literature.

 It would be nice if the authors could consider further proofreading. For instance, the authors may want to replace the typo "nnd" by "and".

Is the study design appropriate and does the work have academic merit?

Yes

Is the work clearly and accurately presented and does it cite the current literature?

Partly

If applicable, is the statistical analysis and its interpretation appropriate?

Not applicable

Are all the source data underlying the results available to ensure full reproducibility?

Yes

Are the conclusions drawn adequately supported by the results?

Partly

Are sufficient details of methods and analysis provided to allow replication by others?

Partly

Reviewer Expertise:

Big data, public health, COVID-19, text mining, social media, data visualisation, data science

I confirm that I have read this submission and believe that I have an appropriate level of expertise to confirm that it is of an acceptable scientific standard, however I have significant reservations, as outlined above.

# Response

**Published:** 2023-11-28

**Authors:** Joao Costa

**Affiliations:** UNESCO IRCAI & Jozef Stefan Institute, Slovenia

We again thank the useful review provided, leading us to improve the content of this paper both in regards to the citations to the related-work and to the details of the study. The time passed between the first submission and this new version also changes the relevance and novelty of the results described, but having in consideration that this is more of a position paper, the content remains useful now that it is better referenced.

# Reviewer report: Reviewer response for version 1

**DOI:** 10.21956/openreseurope.15624.r32819

**Published:** 2023-06-29

**Authors:** Haowen Xu

**Affiliations:** 1 Oak Ridge National Laboratory, Bethel Valley Road, USA

**Overall Comments:**

 This manuscript provides a comprehensive review of big data-driven methods for addressing public health and water resources management problems using text mining and complex data visualization. The contributions of this manuscript include some visions and perspectives of media, science, statistics, and sensing over heterogeneous data sources and text mining algorithms, as well as some discussion regarding the implementation of data-driven methods through real-world examples. At the higher level, the methodology and concepts presented in the manuscript align well with the initiative and trend for building smart cities and urban digital twins. The topic is interesting and covers popular research areas in urban informatics. However, this manuscript covers a very wide spectrum of areas (e.g., global water observatory and COVID-19). I recommend the authors refine their scopes and focus. Even within the areas of global water observatory, there are too many perspectives (e.g., hydroinformatics, collaborative watershed management, urban water systems, public engagement, citizen science, and voluntary geographic information). There are also many different types of water resource management (e.g., surface water, groundwater, watershed planning, urban water supply and distribution system, flood and water hazards resilience/vulnerability, river management, and ecohydrology). The "Global Water Observatory" topic is too generic and broad and needs further refinement.

 The authors could make this manuscript a review paper or an opinion paper that focuses on an urban data-driven digital twin approach to support urban management and use global water observatories and public health as case studies. 

**Detailed Comments:**

**Comment 1:**

 I think the manuscript only partially cited a portion of current literature. As an example, there are many public data sources for water resources data. I came from an urban computational science and hydroinformatics background in the U.S., where the government has open water data initiatives. Many real-time and historical government sensor data, such as the USGS streamflow, USEPA STORET water quality data, and FEMA Hazus flood data, are open to the public. University consortiums, such as CUAHSI and UCAR, provide online data-sharing and model-sharing platforms (e.g., CUAHSI Hydroshare) to share water resources data and simulations produced in academic research. The authors could include a more comprehensive review of the open data sources for both water resources and COVID data. Is there any government open water data initiatives or University consortiums data initiatives within the EU? 

**Comment 2:**

 I would suggest the authors provide a quantitative measure of the trend of different technologies and research areas using Elsevier Scorpous or Google Scholar. These research platforms could provide you with statistics on the literature of a specific research area or contain specific keywords over a few years. A quantified bibliometric analysis is recommended.

**Comment 3:**

 The visualization perspective of the topic is very weak in the current manuscript. There are many review articles that talk about visual computing approaches (information visualization, scientific visualization, and visual analytics) for COVID analysis and water resource management. Please see the example below:

- Leung, C. K., Chen, Y., Hoi, C. S., Shang, S., Wen, Y., & Cuzzocrea, A. (2020, September). Big data visualization and visual analytics of COVID-19 data. In *2020 24th International Conference Information Visualisation (IV)* (pp. 415-420). IEEE. ^1^
- Xu, H., Berres, A., Liu, Y., Allen-Dumas, M. R., & Sanyal, J. (2022). An overview of visualization and visual analytics applications in water resources management. *Environmental Modelling & Software*, 105396. ^2^

**Comment 4:**

 In Figure 1, the authors mentioned the concept of digital twins. However, an important perspective on digital twins' features is missing from the discussion in the current manuscript. It is the "predictive analytics" capability, which differs from an urban digital twin from previous urban or hydrologic information systems (or decision support systems).

**Comment 5:**

 Section V should provide more examples of digital twins in the water resources management sectors. More cases and real-world implementations should be discussed here. Below is an example; more examples/instances should be reviewed and discussed here.

- Alperen, C. I., Artigue, G., Kurtulus, B., Pistre, S., & Johannes, A. (2021, November). A hydrological digital twin by Artificial Neural Networks for flood simulation in Gardon de Sainte-Croix basin, France. In *IOP Conference Series: Earth and Environmental Science* (Vol. 906, No. 1, p. 012112). IOP Publishing ^3^.

**Comment 6:**

 The conclusion should also include some technical challenges and aspects of developing urban digital twins for the EU. As an example, what are potential big data or computing challenges from the cyber-infrastructure or HPC, respectively? Are there any data privacy or data residency concerns across EU countries? 

 I would recommend a minor revision for this manuscript. Overall the manuscript is in good shape and is well organized.

Is the study design appropriate and does the work have academic merit?

Yes

Is the work clearly and accurately presented and does it cite the current literature?

Partly

If applicable, is the statistical analysis and its interpretation appropriate?

Not applicable

Are all the source data underlying the results available to ensure full reproducibility?

Yes

Are the conclusions drawn adequately supported by the results?

Partly

Are sufficient details of methods and analysis provided to allow replication by others?

Partly

Reviewer Expertise:

Urban Digital Twins, Visual Analytics, Information Visualization, Hydroinformatics, Urban Science, Cyber Physical Systems, Watershed Management (with focus on soil erosion and in-stream sediment transport), Intelligent Transportation System, 3D City Modeling

I confirm that I have read this submission and believe that I have an appropriate level of expertise to confirm that it is of an acceptable scientific standard, however I have significant reservations, as outlined above.

## References

[1] : Big Data Visualization and Visual Analytics of COVID-19 Data. *2020 24th International Conference Information Visualisation (IV), Melbourne, Australia* .2020; 10.1109/IV51561.2020.00073 415-420 10.1109/IV51561.2020.00073

[2] : An overview of visualization and visual analytics applications in water resources management. *Environmental Modelling & Software* .2022;153: 10.1016/j.envsoft.2022.105396 10.1016/j.envsoft.2022.105396

[3] : A Hydrological Digital Twin by Artificial Neural Networks for Flood Simulation in Gardon de Sainte-Croix Basin, France. *IOP Conference Series: Earth and Environmental Science* .2021;906(1) : 10.1088/1755-1315/906/1/012112 10.1088/1755-1315/906/1/012112

# Response

**Published:** 2023-11-28

**Authors:** Joao Costa

**Affiliations:** UNESCO IRCAI & Jozef Stefan Institute, Slovenia

We thank the thorough and useful review. We have added the suggested and other citations, and when possible we have exchanged web articles by research papers. Moreover, in the new submitted version of this paper, we have provided some more content regarding open data and visualisations. We have also improved the readability of the paper.
